# Effect of Nano-Y_2_O_3_ on the Microstructure and Properties of WC-Reinforced Ni-Based Composite Surfacing Layer

**DOI:** 10.3390/ma15051665

**Published:** 2022-02-23

**Authors:** Xingyu Ai, Zhengjun Liu, Zongxuan Zou, Zhenyu Wang

**Affiliations:** Department of Material Science and Engineering, Shenyang University of Technology, Shenyang 110870, China; 18809816959@163.com (X.A.); 15942492610@163.com (Z.Z.); wzy1565458906@163.com (Z.W.)

**Keywords:** nano-Y_2_O_3_, WC, nickel-based surfacing layer, microstructure, wear resistance

## Abstract

In this study, a WC-reinforced Ni-based surfacing layer was prepared on Q235 steel plate by plasma arc welding. The effects of nano-Y_2_O_3_ with different contents (0 wt.%, 0.4 wt.%, 0.8 wt.%, 1.2 wt.%, and 1.6 wt.%) on the microstructure, phase composition, microhardness, and wear resistance of the surfacing layer were studied by scanning electron microscope (SEM), energy dispersive spectrometer (EDS), X-ray diffraction (XRD), microhardness test, and pin-on-disk test. The results show that the phase composition of the surfacing layer was γ-Ni, FeNi_3_ solid solution, WC, W_2_C, M_23_C6, M_6_C, Cr_7_C_3_, and other carbides. When the addition of nano-Y_2_O_3_ was 1.2 wt.%, it has a good improvement on microstructure grain refinement and carbide hard-phase increase. Compared with other contents, 1.2 wt.% nano-Y_2_O_3_ surfacing layer has the highest microhardness and the lowest friction coefficient and wear loss. At this time, the wear mechanism is abrasive wear accompanied by slight adhesive wear.

## 1. Introduction

Wear is one of the common failure modes of mechanical parts, and traditional steel materials find increasing difficulty in meeting the demands of special environments [1]. To improve the service life of components, surface engineering techniques are widely used in engineering practice [2,3,4]. Surfacing a wear-resistant layer on components is the most common means, which is of great significance for the safe use of workpieces. Currently, metal matrix composites (MMCs), which can combine the advantages of ceramic and metal materials, are widely used in material surface cladding technology. MMCs are composites based on metals, metal alloys, and intermetallic compounds, which are obtained by adding high-performance reinforcing fibers [5,6], whiskers and particles [7,8], or by the reaction to form reinforcing phases. Metal matrix composites have high strength, stiffness, good thermal stability, wear resistance, creep resistance, and good dimensional stability [9]. At present, the matrix used in MMCs includes the iron matrix, titanium matrix, nickel matrix, etc. [10,11]. Among them, nickel matrix self-melting alloy powder has excellent comprehensive properties and can greatly improve the surface properties of materials [12,13,14,15].

The addition of some ceramic particles to the nickel-based powder enables the surfacing layer to achieve higher hardness, wear resistance, corrosion resistance, etc. [16,17]. WC is a hard phase commonly used in MMCs. WC powder has a high melting point, high hardness, good wear resistance, and good wettability with MMCs. Therefore, WC reinforced nickel-based alloy surfacing layer has been widely used [18,19,20,21]. Acker K V et al. [22] prepared WC/W_2_C carbide-reinforced nickel-based coatings by laser cladding. The results show that both the increase in carbide concentration and the decrease in size are beneficial for wear resistance. Kwon H et al. [23] prepared highly toughened TiC-Ni type cermet using nano-TiC and WC powders. The data show that enhanced toughness is due to solid–solution strengthening of the carbide phase, combined with WC whisker reinforcement.

It is generally believed that rare earth elements have an elemental activity effect, which can segregate impurity elements in the surfacing layer to the grain boundary, inhibit the growth of columnar crystals, refine the microstructure and strengthen the wear resistance of the surfacing layer. The addition of rare earth elements in the surfacing layer can also improve the fluidity and wettability of metal-based composite powder, eliminate defects such as pores, and improve the macro formability of weld. Therefore, the application of rare earth particles to modify the surfacing layer has become a hot spot for current research [13,24,25,26]. Lin et al. [27] found that the optimal addition of Y_2_O_3_ promoted the formation of TiC and TiSi_2_ hard phases, thus improving the microhardness and wear resistance of the cladding layer. Zhao et al. [28] prepared nickel-based coatings with nanoscale La_2_O_3_ by laser melting and found that the addition of La_2_O_3_ significantly refined γ- (Ni, Fe) dendrites, improving the hardness and wear properties of the coatings. Wang et al. [29,30] investigated the microstructural characteristics of laser clad nickel-based alloy coatings with rare earth oxide CeO_2_ or La_2_O_3_. The results show that the microstructure is refined and the wear resistance and corrosion resistance are enhanced after adding rare earth oxide. Li et al. [31] prepared TiB/TiC-reinforced titanium matrix composite coatings on Ti_6_Al_4_V substrates by laser melting. It was shown that the addition of Y_2_O_3_ played a positive role in the wear resistance of the coatings. Yun et al. [32] investigated the effect of different Y_2_O_3_ additives on the wear resistance of hypereutectic Fe-Cr-C hardfacing coatings. The results showed that Y_2_O_3_ could refine the M_7_C_3_ carbide and significantly improve the wear resistance of the coatings. In this paper, nano-Y_2_O_3_ modified nickel-based WC surfacing layers with different contents were prepared on Q235 steel plate by plasma surfacing technology. The effects of nano-Y_2_O_3_ content on macromorphology, microstructure, microhardness, and wear properties of the nickel-based WC (30%) surfacing layer were studied.

## 2. Materials and Methods

**Figure 1:**
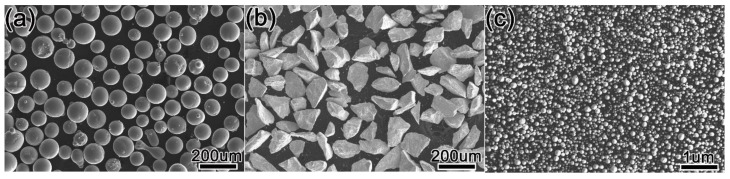
Morphology of the three powders (**a**) Ni60, (**b**) WC, and (**c**) Nano-Y_2_O_3_.

Q235 mild steel was used as the substrate in the present study, which was cut to a dimension of 100 mm × 80 mm × 20 mm by WEDM (Wire Electrical Discharge Machining), and the chemical composition of Q235 is shown in Table 1. Three powders used in the study are spherical Ni60 (150–300 mesh), rhombic WC with 99.9% purity (100–300 mesh), and spherical Y_2_O_3_ with 99.9% purity (Nanoscale). The chemical composition of Ni60 is shown in Table 2, and the morphologies of the three powders are shown in Figure 1.

**Figure 2:**
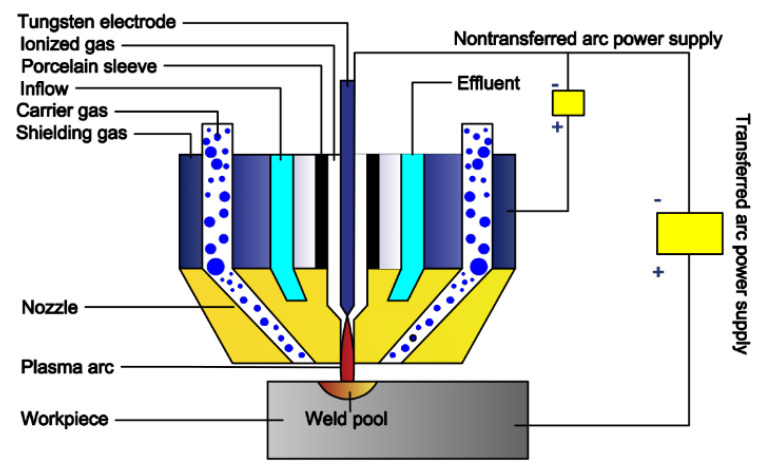
Schematic diagram of powder plasma surfacing equipment.

Before the test, the oxide scale of the substrate was removed with an angle grinder, then the oil stain was removed with acetone, and finally the substrate was sandblasted. Y_2_O_3_ measuring 0 wt.%, 0.4 wt.%, 0.8 wt.%, 1.2 wt.% and 1.6 wt.% was mixed with 30% WC-Ni60 powder by a powder blender. Five different powders were milled by planetary muller for two hours, and the milled powder was placed into a 150 ℃ drying oven for two hours. A plasma surfacing equipment (BX-ZH-400A) was applied for surface overlaying. A schematic diagram of welding equipment is shown in Figure 2. In the present study, the welding current is 120A, the welding speed is 100 mm/min, the powder feeding speed is 15 g/min, the plasma gas flow rate is 4 L/min, the shielding gas flow rate is 10 L/min, and the distance between the nozzle and substrate is 12 mm.

**Figure 3:**
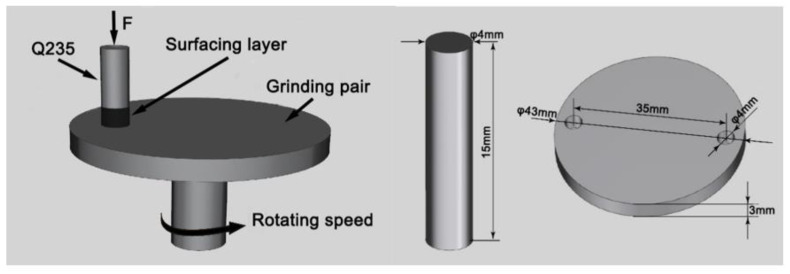
Schematic diagram of the pin-on-disc wear and specimen size.

**Figure 4:**
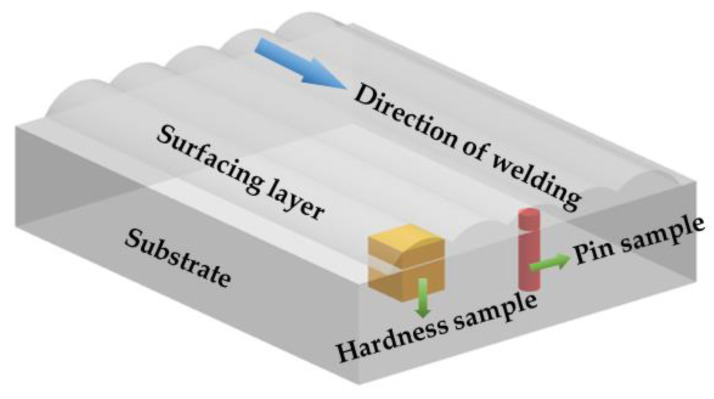
Schematic diagram of sampling location.

S-3400N scanning electron microscope was used to observe the microstructure of the surfacing layer and the wear morphology of the specimens after wear. Xrd-7000 X-ray diffractometer was used to analyze the phase of surfacing layer, and the Cu-kα radial was selected with a working voltage of 40 KV, working current of 30 mA, scanning speed of was 2°/min, and scanning range was 20° to 90°. The Vickers hardness of the surfacing layer was tested by HVS-5 Vickers hardness tester, and the test load was 0.5 kg and the loading time was 15 s. Bearings, gears, sliders, and valves are the most susceptible to wear among mechanical components, and about 80% of the components in the use of mechanical equipment are invalid due to wear [33]. In this experiment, the pin-on-disk wear test is used to simulate metal-to-metal wear under actual working conditions. Friction wear testing machine was used to test the wear resistance of the specimens. The specimen was made into a φ4 × 15 mm round bar, and the thickness of the wear-resistant surfacing layer on the top of the pin is 5 mm. The GCr15 (High-carbon chromium bearing steel) disc with a hardness of 61HRC after quenching and tempering was selected for the grinding pair, and the surface roughness is Ra = 0.8; the chemical composition of GCr15 is shown in Table 3, and the schematic diagram of the pin-on-disc wear and the specimen size is shown in Figure 3. The parameters of the pin-on-disc wear test were as follows: loading force 300 N, rotating speed 100 r/min, time 30 min, and temperature 400 ℃. The wear specimen was weighed before and after the test, and the friction coefficient of the specimen was recorded by the sensor connected with the computer. The sampling positions of the Vickers hardness test and the pin-on-disc wear test are shown in Figure 4.

## 3. Results and Discussion

### 3.1. Effect of Y_2_O_3_ Content on the Formability of Surfacing Layer

**Figure 5:**
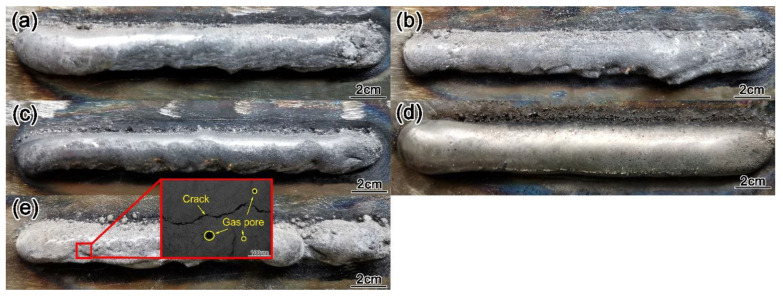
Macro morphology of surfacing layer with different content of Y_2_O_3_ (**a**) 0 wt.%, (**b**) 0.4 wt.%, (**c**) 0.8 wt.%, (**d**) 1.2 wt.%, and (**e**) 1.6 wt.%.

Figure 5 shows the macromorphology of the surfacing layer with different content of Y_2_O_3_. (a 0 wt.%, b 0.4 wt.%, c 0.8 wt.%, d 1.2 wt.%, and e 1.6 wt.%). It can be seen from the figure that as the amount of Y_2_O_3_ added increases from 0 wt.% to 1.2 wt.%, the surface morphology of the surfacing layer gradually becomes flat and smooth, the spatter rate decreases, and the powder can be completely melted. When the amount of Y_2_O_3_ was added to 1.6 wt.%, the surface of the surfacing layer began to have wrinkles, pores, and cracks appear and they formed poorly, indicating that the right amount of Y_2_O_3_ can obtain a well-formed surfacing layer.

**Figure 6:**
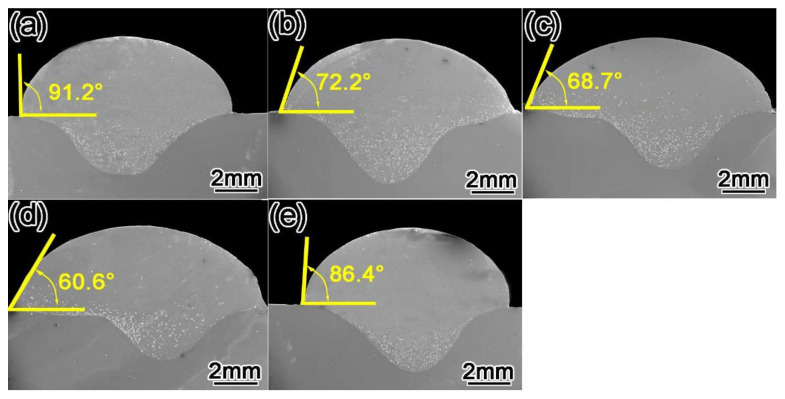
Cross-sectional morphology of surfacing layer with different content of Y_2_O_3_ (**a**) 0 wt.%, (**b**) 0.4 wt.%, (**c**) 0.8 wt.%, (**d**) 1.2 wt.%, and (**e**) 1.6 wt.%.

Figure 6 shows the cross-sectional shape of the surfacing layer perpendicular to the welding direction in which Y_2_O_3_ content is 0 wt.%, 0.4 wt.%, 0.8 wt.%, 1.2 wt.%, and 1.6 wt.%, respectively. It can be seen from the figure that with the increase in Y_2_O_3_ content, the wetting angle decreases first and then increases.

**Figure 7:**
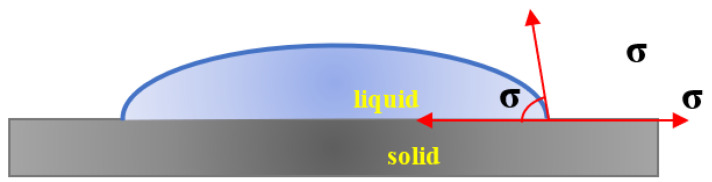
Schematic diagram of wetting.

Wetting refers to the process in which one fluid on the surface of the solid is replaced by another fluid. As shown in Figure 7, basic wetting can be expressed by the young Dupre equation as follows [34].

(1) $$\cos\theta =\left(\sigma_{sg}-\sigma_{sl}\right)/\sigma_{lg}$$

The smaller the wetting angle θ, the better the wettability. Wettability depends on the surface tension of solid σ_sg_, solid–liquid interfacial tension σ_sl_, and liquid surface tension σ_lg_ with respect to the magnitude of these three forces. For liquids, the lower the surface tension, the better the wettability [35,36]. The reason is that Y_2_O_3_ is added to the deposited metal as an oxide dispersed particle. The conductivity and thermal conductivity of the deposited metal decrease with the increase in Y_2_O_3_ content so as to reduce the surface tension of the molten pool metal and reduce the wetting angle, indicating that wettability between the molten pool metal and the base metal will be improved, Therefore, an appropriate amount of Y_2_O_3_ can obtain a surfacing layer with good forming quality.

### 3.2. Analysis of Phase and Microstructure

**Figure 8:**
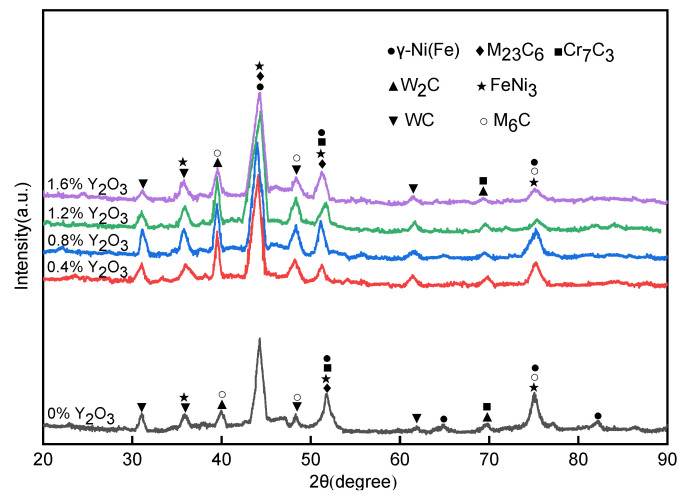
X-ray diffraction pattern of surfacing layer with different Y_2_O_3_ content.

In order to understand the effect of different content of nano-Y_2_O_3_ on the phase evolution of the surfacing layer, the surface of the surfacing layer was analyzed by X-ray diffraction. As can be seen from Figure 8, the main diffraction peak is γ-Ni (Fe), indicating that the γ-Ni (Fe) solid solution is the matrix phase of the surfacing layer. An XRD spectrum shows that carbides such as WC, W_2_C, M_23_C_6_, M_6_C, Cr_7_C_3_, and FeNi_3_ compounds also exist in the surfacing layer. Overall, the presence or absence of Y_2_O_3_ has a small effect on the phase composition of the surfacing layer, but it can be observed that with the addition of nano-Y_2_O_3_, the peak strength of carbide increases. This is because nano-Y_2_O_3_ does not change the phase composition, but will act as heterogeneous nuclear particles to promote the formation of carbide [37].

**Figure 9:**
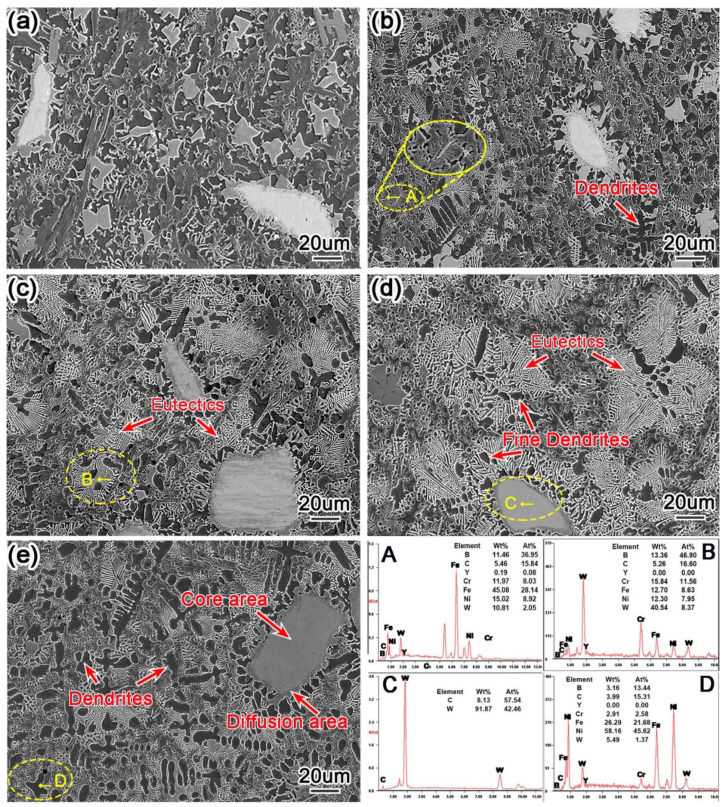
SEM images of Y_2_O_3_-modified Surfacing layer along with EDS maps (**a**) 0 wt.%, (**b**) 0.4 wt.%, (**c**) 0.8 wt.%, (**d**) 1.2 wt.%, and (**e**) 1.6 wt.%.

Figure 9 shows scanning electron microscope (SEM) and energy dispersive spectrometer (EDS) images of nano-Y_2_O_3_ modified surfacing layers with different contents. It can be observed from the figure that the microstructure of the surfacing layer is composed of dendrites and eutectic compounds. Combined with EDS and X-ray diffraction (XRD) analysis, region A contains M_6_C carbide, region B contains composite carbide with WC, W_2_C, region C is mainly primary dendrites γ-Ni, region D comprises gray-white particles, and the main elements are W and C and the atomic ratio ≈ 1:1. It can be judged that granular matter is unfused WC.

When the addition of Y_2_O_3_ is 0.4 wt.%, the microstructure of the surfacing layer begins to change, and white lamellar eutectic carbide can be observed to begin to appear, with more grey eutectic tissue around it. When Y_2_O_3_ content increases to 0.8 wt.%, the distribution of microstructure is relatively dense, the white eutectic carbides around unfused WC increase obviously, and a large number of dendrites are gathered around these carbides. By observing WC in Figure 9e, it can be observed that there is a gray–white area in the center of the WC, which is called the core area. In the periphery of WC, there is a silvery gray area with a thickness of about 3 um, which is called the melting diffusion area. According to the relevant carbide melting mechanism [38], when WC particles enter the melting pool, the outer layer of larger particles melts under the action of high-temperature plasma arc and attaches around the core area. With the addition of nano Y_2_O_3_ reaching 1.2 wt.%, as the core of heterogeneous nucleation [37], lamellar eutectic carbides begin to increase, and these eutectic carbides attach to the unfused WC core area and grow around it. Although the presence of Y was not detected in XRD analysis, a small amount of Y was found in EDS analysis.

Studies have shown that high melting point rare nano-Y_2_O_3_ has difficulty in completely melting in the process of plasma surfacing, and the incompletely melted Y_2_O_3_ powder in the surfacing layer will more easily become the core of heterogeneous phase nucleation, which is conducive to improving nucleation rates and promoting nonspontaneous nucleation so as to refine grains [39]. In this study, only the addition of Y_2_O_3_ was changed, and the content of other elements remained unchanged, but the carbides significantly increased, as seen in Figure 9, which confirmed the above view from the side, and the increased carbides were nucleated carbides. At the same time, nano-Y_2_O_3_ will decompose into Y atoms with large atomic radius and good chemical activity at high temperature, and Y atoms will gather at the grain boundary, resulting in a drag effect on the grain boundary and inhibiting grain growth [40,41]. When the amount of Y_2_O_3_ added to the surfacing layer increases to 1.6 wt.%, the microstructure is observed not to change much, but the eutectic carbides start to decrease and dendrite size increases. This is because the addition of excessive nano-Y_2_O_3_ reduces the fluidity of the molten pool, slows down the convection speed, makes it difficult to discharge inclusions and pores, reduces the cooling speed of the molten pool, and increases the size of these dendrites for a long time.

**Figure 10:**
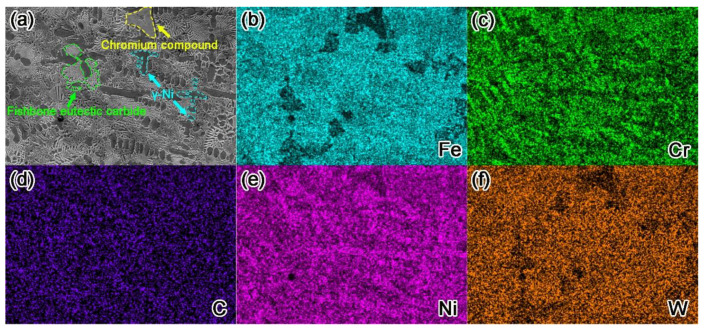
SEM image of the microstructure of the surfacing layer (**a**) and the corresponding energy dispersive spectroscopy (EDS) maps (**b**–**f**).

Figure 10 shows the element distribution of the microstructure of the surfacing layer. The areas with bright colors in surface scanning are the enrichment area of the element, and the areas with dark colors are the depleted area of the element. It can be observed intuitively from the figure that the dendrite region is rich in Fe, Ni, and W elements, indicating that this region is mainly composed of γ- Ni matrix phase. The massive area is mainly composed of Cr elements, with low contents of Fe, Ni, and W elements, which should be chromium compounds. The white fishbone microstructure and massive area have more Cr and C elements, and there are Ni, Fe, and W elements, indicating that the microstructure in this area is mostly carbide, which is basically consistent with the previous EDS energy spectrum analysis results.

### 3.3. Microhardness and Wear Resistance Analysis of the Surfacing Layer

**Figure 11:**
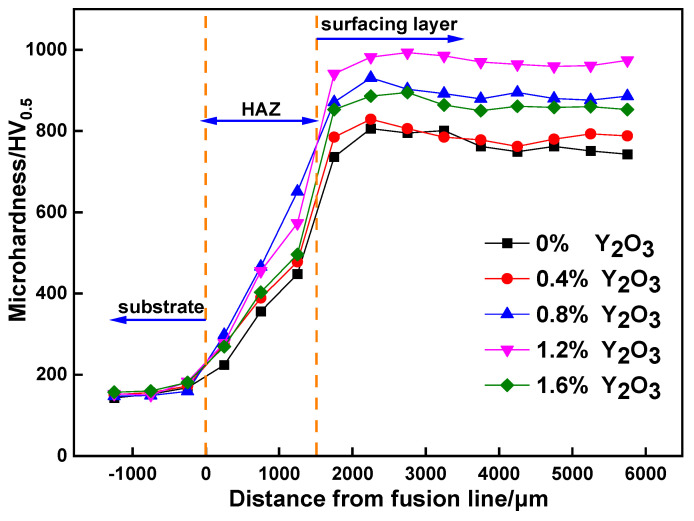
Microhardness of surfacing layer with different Y_2_O_3_ content.

Figure 11 shows the microhardness curves of the surfacing layer with different Y_2_O_3_ content. From the figure, it can be observed that the microhardness of the surfacing layer has a significant increase compared to the base metal, while the microhardness of the heat affected zone (HAZ) is lower than that of the surfacing layer due to the dilution effect of the base metal. Compared with the surfacing layer without Y_2_O_3_, the microhardness of the surfacing layer with Y_2_O_3_ increased significantly. When the addition of Y_2_O_3_ is 0.4 wt.%, the microhardness of the surfacing layer does not increase significantly. With the addition of Y_2_O_3_ increasing to 1.2 wt.%, the average microhardness of the surfacing layer reaches the maximum, which is 970HV_0.5_, about 6.26 times of the base metal hardness, 283HV_0.5_ higher than the surfacing layer without Y_2_O_3_, and the microhardness value of each point of the surfacing layer is the most uniform under this content.

Some studies show that the transformation of hardness originates from the formation of interdendritic carbides and eutectic structures [42]. Corresponding to Figure 9, it can be observed that the addition of Y_2_O_3_ promotes a uniform dispersion of carbides in the surfacing layer during the formation process, and Y_2_O_3_ with a high melting point, as a nucleating agent, effectively refines the microstructure. Under the combined action of dispersion strengthening and fine grain strengthening, the hardness of the surfacing layer has been significantly improved. When the addition of Y_2_O_3_ is 1.6 wt.%, the microhardness of the surfacing layer decreases obviously. At this time, when Y_2_O_3_ is added excessively, the flow performance of the surfacing layer decreases, inclusions are generated in the surfacing layer, gas is difficult to escape, pores and other defects are generated, and the cooling time of the molten pool increases, which affects the microstructure of the surfacing layer so as to reduce the hardness of the surfacing layer.

**Figure 12:**
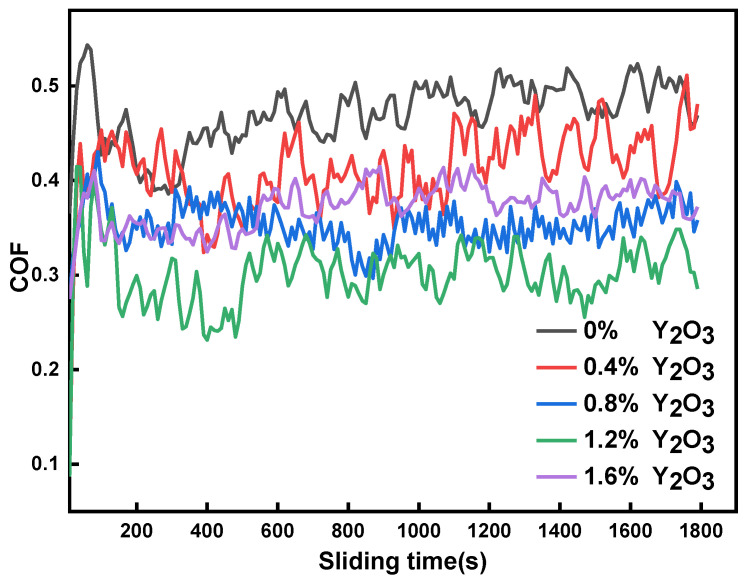
Friction coefficient curve of the modified surfacing layer with different contents of Y_2_O_3_.

Figure 12 shows the friction coefficient of the modified surfacing layer with different contents of Y_2_O_3_ at room temperature with GCr15 as a grinding pair is worn for 30 min. It can be observed from the figure that the friction coefficient of the surfacing layer modified with different content of Y_2_O_3_ varies greatly. In the initial stage of the experiment, the friction coefficient fluctuates greatly, which is caused by the machined surface roughness. Under the action of load, the contact surfaces mesh with each other. After the initial running in stage, the wear debris will fill the bonding interface, the surfacing layer will enter a stable wear stage, and the fluctuation range of the friction coefficient curve tends to be stable. After adding Y_2_O_3_, the friction coefficient of the surfacing layer decreases significantly, which can improve the wear performance of the surfacing layer. When the content of Y_2_O_3_ is 1.2 wt.%, the friction coefficient of the surfacing layer is the smallest, about 0.3092. It shows that the addition of an appropriate amount of Y_2_O_3_ can increase and evenly distribute the hard phase in the surfacing layer and form a wear-resistant skeleton on the surface of the surfacing layer, effectively improving the wear resistance of the surfacing layer. When the addition amount of Y_2_O_3_ increases to 1.6 wt.%, it can be observed that the friction coefficient begins to increase. Due to the fact that the content of Y_2_O_3_ added is too high and Y_2_O_3_ is difficult to melt, it will greatly affect the fluidity of the weld pool in the surfacing process, resulting in uneven distribution of hard phase, and the unmelted Y_2_O_3_ and other elements in the weld pool form harmful inclusions. At the same time, it may also produce defects such as pores, deteriorate the microstructure of the surfacing layer, increase the friction coefficient, and reduce the wear resistance of the surfacing layer.

**Figure 13:**
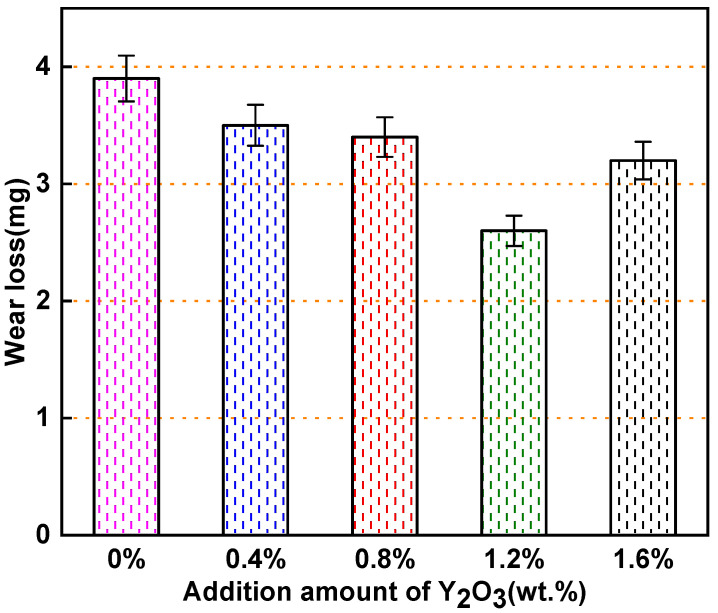
Wear loss of Y2O3 modified surfacing layer with different content.

Figure 13 shows the wear weight loss of the Y_2_O_3_ modified surfacing layer with different content worn for 30 min at room temperature. It can be observed that the addition of Y_2_O_3_ can improve the wear performance of the surfacing layer. With the increase in Y_2_O_3_ addition, the wear first decreases and then increases. When the addition amount of Y_2_O_3_ is 1.2 wt.% The wear performance of the surfacing layer is the best and the wear amount is the lowest, which constitutes only 2.6 mg. This indicates that the wear resistance of the Y_2_O_3_ composite surfacing layer with 1.2 wt.% addition is 1.5-times higher than that of the nickel-based tungsten carbide surfacing layer.

**Figure 14:**
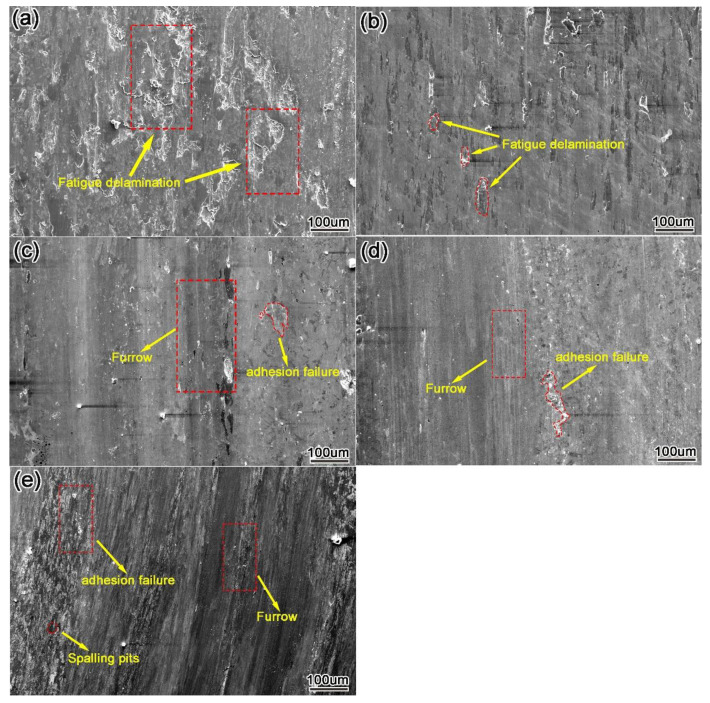
Wear morphology of modified surfacing layer with different contents of Y_2_O_3_ (**a**) 0 wt.%, (**b**) 0.4 wt.%, (**c**) 0.8 wt.%, (**d**) 1.2 wt.%, and (**e**) 1.6 wt.%.

Figure 14 shows the wear morphology of the Y_2_O_3_ modified nickel-based tungsten carbide surfacing layer after 30 min of wear at room temperature. The surface of the surfacing layer without the addition of Y_2_O_3_ (Figure 14a) shows a large amount of fatigue delamination. With 0.4 wt.% Y_2_O_3_ (Figure 14b), a small amount of fatigue delamination is visible on the surface of the surfacing layer, and the wear mechanism is delamination wear. With the addition of 0.8 wt.% Y_2_O_3_ (Figure 14c), furrows with different depths can be observed on the surface of the surfacing layer, accompanied by a small amount of white and bright oxidized wear debris. The wear mechanism is mainly abrasive wear, accompanied by oxidative wear and adhesive wear.

After the addition of 1.2 wt.% Y_2_O_3_ (Figure 14d), furrows on the surface of the surfacing layer are shallow, the plastic deformation characteristics are not obvious, and there are still peeling pits and oxide debris caused by fatigue. This is because an appropriate amount of Y_2_O_3_ can uniformly disperse the hard phase in the surfacing layer, refine the grain, improve the ability to resist dislocation slip and promote γ- Ni matrix forms a wear-resistant skeleton with excellent wear resistance, which can effectively protect the surfacing layer from wear damage when cutting wear occurs between wear debris and matrix. After the addition of 1.6 wt.% Y_2_O_3_ (Figure 14e), adhesive wear marks and peeling pits appeared on the surface. Excessive Y_2_O_3_ will reduce the fluidity of the melt pool and the refractory Y_2_O_3_ will form inclusions with other elements in the melt pool and cause a large number of pores and other defects in the surfacing layer, resulting in a reduction in the hardness of the surfacing layer and affecting the wear performance of the surfacing layer.

## 4. Conclusions

In this study, the Y_2_O_3_-modified nickel-based WC surfacing layer was successfully prepared by adding nano-Y_2_O_3_. The effects of Y_2_O_3_ on the macro morphology, phase composition, microstructure, hardness, and wear resistance on the surfacing layer of the alloy have been systematically studied. The main conclusions are as follows:

1. By adding different contents of Y_2_O_3_ into the 30 wt.% WC-Ni based alloy surfacing layer, it can be observed that the addition of Y_2_O_3_ with a content of 1.2% can effectively reduce the wetting angle and improve weld formability. However, an excessive amount of Y_2_O_3_ can cause welding defects.
2. The formation of phase was found to be independent of the amount of RE addition, and its main phase is γ-Ni (Fe), FeNi_3_ solid solution. Carbides were formed with various morphology, namely, WC, W_2_C, M_23_C_6_, M_6_C, and Cr_7_C_3_.
3. An appropriate amount of Y_2_O_3_ can significantly refine microstructure and increase the content of eutectic carbides. However, excessive Y_2_O_3_ will cause coarse microstructure and reduce the content of carbides.
4. The addition of nano-Y_2_O_3_ significantly improved the average hardness of the surfacing layers. With 1.2 wt.% nano-Y_2_O_3_, the surfacing layer reaches the maximum average of Vickers hardness, 970HV_0.5_, which is about six-times harder than the base metal.
5. According to the pin-on-disk wear experiment results, an appropriate amount of nano-Y_2_O_3_ at room temperature can reduce the coefficient of friction (COF) and wear of the surfacing layer. The minimum wear of the surfacing layer containing 1.2 wt.% nano-Y_2_O_3_ was 2.6 mg, whereas the minimum COF was approximately 0.3092. The wear mechanism is mainly abrasive wear, accompanied by slight adhesive wear.

## Figures and Tables

**Table 1:** Chemical composition of Q235 low carbon steel (wt.%).

Element	C	Mn	Si	S	P	Fe
Content	0.12~0.22	0.30~0.70	≤0.37	≤0.050	≤0.045	bal.

**Table 2:** Chemical composition of the Ni60 alloy (wt.%).

Element	C	B	Si	Cr	Fe	Ni
Content	0.7~1.0	3.0~4.5	3.5~4.5	15~17	≤8	Bal.

**Table 3:** Chemical composition of GCr15 (wt.%).

Element	C	Si	Mn	Cr	Mo	S	P	Ni	Cu	Ni + Cu	Fe
Content	0.95~1.05	0.15~0.35	0.25~0.45	1.40~1.65	≤0.10	≤0.025	≤0.025	≤0.30	≤0.25	≤0.50	bal.

## Data Availability

All the data and results supporting this research paper are already presented within this publication.
